# Ramsay Hunt syndrome

**DOI:** 10.11604/pamj.2019.34.201.19207

**Published:** 2019-12-16

**Authors:** Yaseer Muhammad Tareq Khan, Nishat Fatema

**Affiliations:** 1 Department of Acute Internal Medicine, East Surrey Hospital, Surrey and Sussex Healthcare NHS Trust, Canada Ave, Redhill RH1 5RH, United Kingdom; 2 Department of Obstetrics and Gynecology, Imperial Hospital Limited, Chittagong, Bangladesh

**Keywords:** Ramsay Hunt syndrome, herpes zoster oticus, facial nerve palsy

## Image in medicine

**Figure 1:**
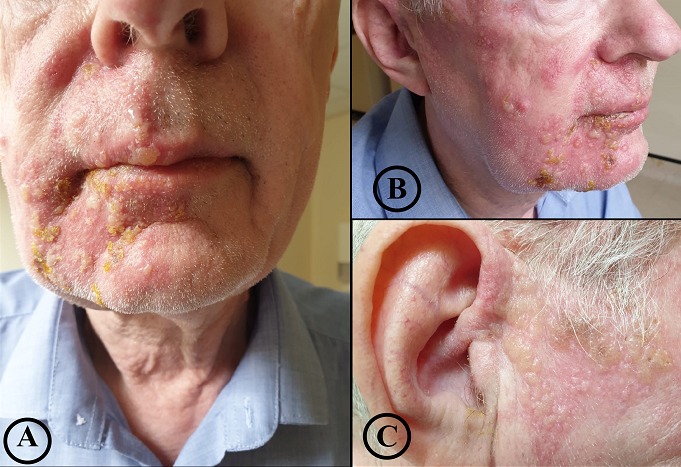
A) ipsilateral (right) mild facial palsy; B) multiple vesicular lesions over right side of the face; C) preauricular vesicular rash

A 63-year-old man presented to our hospital with 2 days history of right-sided earache and numbness followed by the development of vesicular rash involving the right side of the face. There was no history of headache, tinnitus, giddiness or hearing impairment. Neurological examination revealed right-sided mild lower motor neuron type facial palsy (A) and vesicular rash in the distribution of maxillary and mandibular branch of trigeminal nerve without any sensorineural deafness (B, C). Routine all laboratory examinations including retroviral screening were normal. A clinical diagnosis of the Ramsay Hunt syndrome (RHS) was considered based on earache, facial paralysis and typical dermatomal distribution of rash. He was started with oral valaciclovir 1gm three times daily (TDS) for 7 days and Tab prednisolone 60mg daily for 5 days. Two days after initiation of the treatment vesicular lesions disappeared but neurological examination revealed right-sided grade IV facial nerve palsy. Ramsay Hunt syndrome or Herpes Zoster Oticus is characterized by reactivation of latent varicella zoster virus in the geniculate ganglion and subsequent spread to cranial nerve. The diagnosis is mainly clinical. The facial paralysis seen in Ramsay Hunt syndrome is often more severe with the increased rate of late neural denervation and decrease the chance of complete recovery. Figure 1

